# Supplementary material for: Multi‐dataset identification of innovative feature genes and molecular mechanisms in keratoconus
Source: J Cell Mol Med. 2024 Sep 19;28(18):e70079. doi: 10.1111/jcmm.70079 (PMC11412914; doi:10.1111/jcmm.70079)
Supplement: Supplementary file 2 — Material S2. [file JCMM-28-e70079-s002.zip › 7.GSEA analysis/1.ARL11/KEGG_CYSTEINE_AND_METHIONINE_METABOLISM.html]

Details for gene set KEGG\_CYSTEINE\_AND\_METHIONINE\_METABOLISM[GSEA]

|  || Dataset | ARL11.group.cls#h\_versus\_l |
| Phenotype | group.cls#h\_versus\_l |
| Upregulated in class | h |
| GeneSet | KEGG\_CYSTEINE\_AND\_METHIONINE\_METABOLISM |
| Enrichment Score (ES) | 0.18378964 |
| Normalized Enrichment Score (NES) | 0.7991424 |
| Nominal p-value | 0.76687115 |
| FDR q-value | 1.0 |
| FWER p-Value | 1.0 |
Table: GSEA Results Summary

  

Fig 1: Enrichment plot: KEGG\_CYSTEINE\_AND\_METHIONINE\_METABOLISM      
 Profile of the Running ES Score & Positions of GeneSet Members on the Rank Ordered List

  

| SYMBOL | TITLE | RANK IN GENE LIST | RANK METRIC SCORE | RUNNING ES | CORE ENRICHMENT || 1 | BHMT | na | 1693 | 0.320 | 0.0845 | Yes |
| 2 | IL4I1 | na | 4200 | 0.194 | 0.0928 | Yes |
| 3 | MTAP | na | 6223 | 0.131 | 0.0891 | Yes |
| 4 | MAT2A | na | 6269 | 0.130 | 0.1419 | Yes |
| 5 | LDHAL6A | na | 6578 | 0.122 | 0.1838 | Yes |
| 6 | MTR | na | 8172 | 0.089 | 0.1749 | No |
| 7 | GOT2 | na | 9613 | 0.066 | 0.1607 | No |
| 8 | MAT1A | na | 10045 | 0.060 | 0.1731 | No |
| 9 | LDHC | na | 13378 | 0.024 | 0.0870 | No |
| 10 | DNMT3L | na | 14196 | 0.017 | 0.0707 | No |
| 11 | MPST | na | 15842 | 0.005 | 0.0253 | No |
| 12 | AMD1 | na | 16846 | -0.002 | -0.0027 | No |
| 13 | CDO1 | na | 17122 | -0.004 | -0.0090 | No |
| 14 | AHCYL1 | na | 17508 | -0.007 | -0.0172 | No |
| 15 | DNMT3A | na | 19111 | -0.018 | -0.0560 | No |
| 16 | GOT1 | na | 19508 | -0.021 | -0.0589 | No |
| 17 | SRM | na | 20072 | -0.024 | -0.0651 | No |
| 18 | SMS | na | 21570 | -0.036 | -0.0933 | No |
| 19 | DNMT1 | na | 21682 | -0.037 | -0.0813 | No |
| 20 | AHCY | na | 22062 | -0.040 | -0.0753 | No |
| 21 | ADI1 | na | 22077 | -0.041 | -0.0588 | No |
| 22 | TRDMT1 | na | 22387 | -0.044 | -0.0495 | No |
| 23 | APIP | na | 22630 | -0.047 | -0.0371 | No |
| 24 | LDHB | na | 22665 | -0.047 | -0.0186 | No |
| 25 | MAT2B | na | 22724 | -0.047 | -0.0006 | No |
| 26 | SDS | na | 22931 | -0.049 | 0.0140 | No |
| 27 | DNMT3B | na | 23719 | -0.059 | 0.0158 | No |
| 28 | ENOPH1 | na | 24152 | -0.064 | 0.0302 | No |
| 29 | CTH | na | 24353 | -0.067 | 0.0523 | No |
| 30 | AHCYL2 | na | 24682 | -0.072 | 0.0727 | No |
| 31 | LDHA | na | 24699 | -0.072 | 0.1022 | No |
| 32 | TAT | na | 24898 | -0.075 | 0.1276 | No |
| 33 | CBS | na | 28775 | -0.151 | 0.0789 | No |
| 34 | LDHAL6B | na | 31068 | -0.223 | 0.1055 | No |
Table: GSEA details [plain text format]

  

Fig 2: KEGG\_CYSTEINE\_AND\_METHIONINE\_METABOLISM      
 Blue-Pink O' Gram in the Space of the Analyzed GeneSet

  

Fig 3: KEGG\_CYSTEINE\_AND\_METHIONINE\_METABOLISM: Random ES distribution      
 Gene set null distribution of ES for **KEGG\_CYSTEINE\_AND\_METHIONINE\_METABOLISM**
